# Composite Photocatalysts with Fe, Co, and Ni Oxides on Supports with Tetracoordinated Ti Embedded into Aluminosilicate Gel during Zeolite Y Synthesis

**DOI:** 10.3390/gels10020129

**Published:** 2024-02-05

**Authors:** Gabriela Petcu, Elena Maria Anghel, Irina Atkinson, Daniela C. Culita, Nicoleta G. Apostol, Andrei Kuncser, Florica Papa, Adriana Baran, Jean-Luc Blin, Viorica Parvulescu

**Affiliations:** 1 Institute of Physical Chemistry “Ilie Murgulescu” of the Romanian Academy, 202 Splaiul Independentei, 060021 Bucharest, Romania; manghel@icf.ro (E.M.A.); iatkinson@icf.ro (I.A.); dculita@icf.ro (D.C.C.); floricapapa@gmail.com (F.P.); adibaran@gmail.com (A.B.); 2 National Institute of Materials Physics, Atomistilor 405A, 077125 Magurele, Romania; nicoleta.apostol@infim.ro (N.G.A.); andrei.kuncser@infim.ro (A.K.); 3 Faculty of Sciences and Technology, University of Lorraine, CNRS, L2CM, F-54000 Nancy, France; jean-luc.blin@univ-lorraine.fr

**Keywords:** Ti-aluminosilicate gel, Ti-zeolite Y, Fe, Co, Ni/Ti-zeolite Y, cefuroxime degradation, photocatalysis, scavenger experiments

## Abstract

Ti-aluminosilicate gels were used as supports for the immobilization of Fe, Co, and Ni oxides (5%) by impregnation and synthesis of efficient photocatalysts for the degradation of β-lactam antibiotics from water. Titanium oxide (1 and 2%) was incorporated into the zeolite network by modifying the gel during the zeolitization process. The formation of the zeolite Y structure and its microporous structure were evidenced by X-ray diffraction and N_2_ physisorption. The structure, composition, reduction, and optical properties were studied by X-ray diffraction, H_2_-TPR, XPS, Raman, photoluminescence, and UV–Vis spectroscopy. The obtained results indicated a zeolite Y structure for all photocatalysts with tetracoordinated Ti^4+^ sites. The second transitional metals supported by the post-synthesis method were obtained in various forms, such as oxides and/or in the metallic state. A red shift of the absorption edge was observed in the UV–Vis spectra of photocatalysts upon the addition of Fe, Co, or Ni species. The photocatalytic performances were evaluated for the degradation of cefuroxime in water under visible light irradiation. The best results were obtained for iron-immobilized photocatalysts. Scavenger experiments explained the photocatalytic results and their mechanisms. A different contribution of the active species to the photocatalytic reactions was evidenced.

## 1. Introduction

Zeolites are support materials widely used for hybrid adsorbents and photocatalyst applications [1,2,3,4,5,6,7] due to their adjustable properties such as high surface area, adsorption, environmentally friendly nature, hydrophobic/hydrophilic surface, and abundant acid/base sites. In photocatalytic reactions, the zeolite acts as a host for the semiconductors or other photoactive guests, and acid/base sites can reduce electron and hole recombination [1]. Thus, the disadvantages of TiO_2_ nanoparticles (the most commonly used semiconductor in photocatalysis), such as their low surface area, poor affinity for hydrophobic organic pollutants, agglomeration tendency, and light scattering, can be overcome [1]. In addition, to improve the separation of photogenerated charge carriers, the zeolite was doped with metal/non-metal or by introducing other semiconductors with a narrow band gap. The most used zeolites are zeolite Y [4,8,9], zeolite 5A [1,3,10,11], and natural zeolites [12,13]. The previous results [1,14,15,16] reported that the efficiency of TiO_2_/zeolite photocatalysts was optimized by an adequate choice of the zeolite type with a certain Si/Al molar ratio, specific surface area, and hydrophobicity. Also, among isolated metal oxide moieties with tetrahedral coordination geometry embedded in heteroatom-containing zeolites, titanium oxide has attracted considerable attention. The photocatalytic activity and selectivity of tetracoordinated Ti^4+^ species from the framework of titanium silicalite-1 (TS-1), which is the most representative one of the “single-site heterogeneous catalysts”, are much higher than those of octahedrally coordinated Ti in the TiO_2_ semiconductor [17]. The mechanism proposed for the photocatalytic reactions is based on the ligand-to-metal charge transfer (LMCT) process (Ti^4+^−O^2−^ $\underset{\to }{hv}$ Ti^3+^−O^−^). However, TS-1 based photocatalysts are relatively less developed, and the largest limitation is the content of the framework Ti. Much of the relevant research was focused on zeolite synthesis with higher tetra-coordinated Ti in the framework [18,19]. Mesoporous TS-1 with a high Ti active site (Ti/(Si + Ti) > 5%) has been successfully synthesized by using the combined (sol–gel/dry gel conversion) method [19]. Thus, the first step was to obtain amorphous SiO_2_-TiO_2_ solids with Si–O–Ti bonds by the sol–gel method. These mixed oxides were impregnated with a tetrapropylammonium hydroxide (TPAOH) solution and hydrothermally treated to obtain more Ti in TS-1.

The first studies regarding the introduction of titanium into the zeolite Y framework by direct synthesis and the effect of Ti concentration on the zeolite structure were carried out by our group [20,21]. Zeolite Y is a faujasite molecular sieve with 7.35 Å diameter pores and a three-dimensional pore structure. The basic structural units are sodalite cages that form supercages that host spheres with high diameters (11.24 Å) [1]. The preservation of the zeolitic network was obtained for 1 and 2% TiO_2_, and its partial disruption was observed for 5% TiO_2_. The synthesis of Ti-Y and the effect of titanium concentration on the formation of the zeolite Y structure were evaluated by freezing the sol–gel phases at various periods [21].

In photocatalytic reactions, Ti sites possess strong adsorption capacity for the activated reactant molecules, and the coordinated O sites can accept reaction intermediates. Since the TiO_2_/zeolite composites exhibit high photocatalytic activity only under UV light irradiation, many approaches have sought to expand the field of applicability of these photocatalysts to visible light by doping a small amount of noble and transition metals [22,23,24,25,26,27,28]. A special interest was evidenced in noble metal nanoparticles (Pt, Pd, Au, and Ag) because of their plasmonic properties [8,9,29,30]. The combination of noble metals with another cheaper and more abundant transition metal was also studied [25]. Among the transition metals whose ions are capable of modifying the optoelectronic properties of TiO_2_, the following stood out: V, Cr, Fe, Co, Ni, Cu, and Zn [22,23,24,26,27,28,31]. The immobilization of these metals on TiO_2_/zeolite created active species, modified the surface properties, promoted photoinduced carrier separation, and consequently enhanced the photocatalytic activity. The photocatalytic degradation mechanism of zeolite-based photocatalysts can vary for different organic compounds due to their sensitization effect. For instance, our previous results evidenced the high activity of Co–Ti- or Ni–Ti-supported photocatalysts on zeolite Y, with microporous and hierarchical structures, in amoxicillin photodegradation [32]. The degradation mechanism of amoxicillin (β-lactam antibiotic) under UV and visible light irradiation, investigated in the presence of scavengers, evidenced the effect of metal species interaction and zeolite support. Hence, for practical environmental applications, the zeolite properties, supported metals, pollutant type, and experiment factors play vital roles in the photodegradation process. In this regard, the effect of reaction conditions such as pH, concentration of photocatalysts, and pollutants on degradation efficiency requires further investigation. Therefore, the results obtained so far sustain the performance of zeolite-based photocatalysts in the degradation of organic pollutants and their use in practical environmental applications.

Here, Fe, Co, and Ni/Ti-containing zeolite Y photocatalysts were obtained, characterized, and utilized in the degradation of cefuroxime (β-lactam antibiotic) from water. Cefuroxime (CFX) was selected as the representative of the second generation of cephalosporin, widely used in medicine for the treatment of a broad range of infectious bacteria [33,34,35]. CFX antibiotic was frequently detected in wastewater, surface water, and soil [33,34,35,36] and had the highest persistence among the tested cephalosporins in wastewater effluent [36]. Scavenger experiments were performed to deepen the photodegradation mechanism. Titanium was immobilized, as tetrahedral coordinated Ti in the zeolite Y framework, during the sol–gel process from the zeolite synthesis. Subsequently, Fe, Co, and Ni oxides (5%) were immobilized on the prepared Ti-containing zeolite Y support by the impregnation method. X-ray diffraction, N_2_ physisorption, H_2_-TPR, XPS, Raman, UV–Vis, and photoluminescence spectroscopy were used to investigate the structure, texture, composition, configuration, and optoelectronics properties of the photocatalysts. The results obtained in this study, both for titanium and second 3d metal species (Fe, Co, and Ni), were compared with those obtained for similar photocatalysts with titanium immobilized on zeolite Y by impregnation [20,32]. This work provided an effective strategy to address the deactivation problem of TiO_2_-based photocatalysts by Ti leaching, thus increasing the chances of reusing the photocatalytic materials for wastewater decontamination. Besides this, the proposed materials also present other advantages in photocatalysis, such as high adsorption capacity, very good dispersion of photocatalytically active species, increasing the contact area between active sites and pollutants, and activity in the visible area. All these properties have been discussed in the present paper.

## 2. Results and Discussion

### 2.1. Structural, Morphological and Textural Properties

#### 2.1.1. X-ray Diffraction

**Figure 1:**
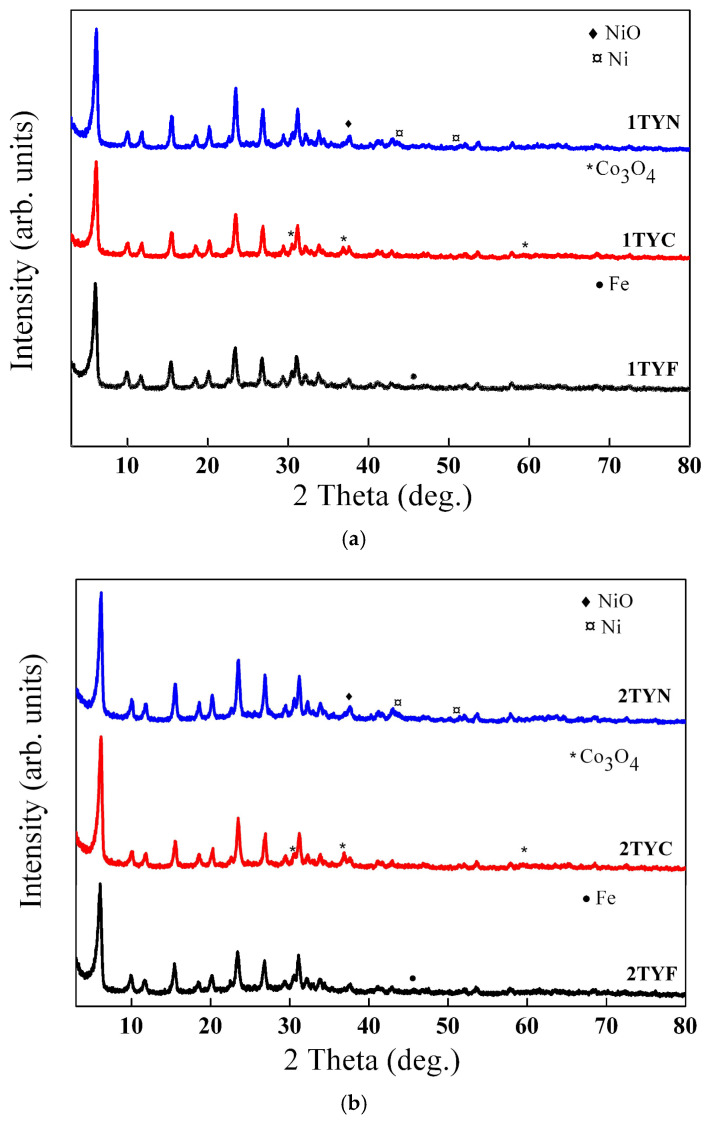
XRD patterns of the (**a**) 1TY(F/C/N) samples and (**b**) 2TY(F/C/N) samples.

Structural analysis of zeolites was performed using X-ray diffraction (XRD) and transmission electron microscopy (TEM). The XRD patterns of the samples with Fe, Co, and Ni supported on Ti-zeolite Y are presented in Figure 1. The results mainly indicate the characteristic peaks of faujasite Y (PDF card no. 00-038-0239). It means that the addition of 3d metal oxides, but especially the incorporation of titanium into the aluminosilicate gel framework, did not affect the obtaining of the crystalline structure of zeolite Y. It was proven in studies previously reported by our group [20,21] that the proposed synthesis method succeeds in obtaining titanium-aluminosilicate with the Y faujasite structure for TiO_2_ concentrations lower than 5%.

In the case of samples modified with cobalt oxide, the XRD results show the formation of the Co_3_O_4_ crystalline phase (PDF card no. 00-042-1467). Thus, the peaks located at 31.19°, 36.84°, and 59.31° were attributed to the Co_3_O_4_ crystal faces (220), (311), and (511), respectively. Modification of Ti-zeolite Y supports with nickel oxide led to the appearance of several discrete peaks in the XRD patterns at 37.52°, 46.76°, 63.85°, and 76.12° assigned to (111), (200), (220), and (311) planes of bunsenite NiO (PDF card no. 00-047-1049). Besides these, the XRD results also indicate the formation of metallic nickel (PDF card no. 00-901-3028). It is sustained by the presence of the peaks that appear at 43.55° and 51.42° and correspond to the (111) and (200) planes of nickel in metallic form. Meanwhile, for the samples modified with iron oxide, the XRD results did not show the presence of any diffraction peak characteristic of crystalline iron oxide forms, although an experimental concentration of 3.04% was determined by XRF measurements. It is the result of small iron oxide crystallites with a high dispersion, which does not allow their detection by X-ray diffraction. However, a slight signal seems to be obtained at 45.58°, suggesting the formation of metallic iron (PDF card no. 00-900-0669). The corresponding X-ray diffractograms of the unmodified Ti-zeolite Y supports (Figure S1) were used as a reference.

#### 2.1.2. TEM Investigation

**Figure 2:**
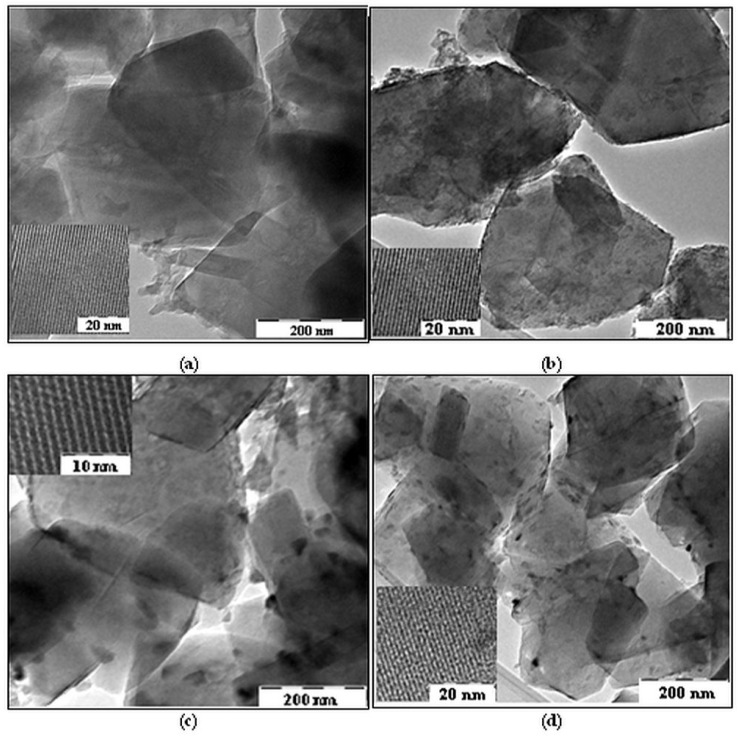
TEM images of (**a**) 1TY, (**b**) 1TYF, (**c**) 1TYC, and (**d**) 1TYN samples.

TEM images show (Figure 2) that metal oxides are deposited on the zeolite crystals, forming islands of nanometric dimensions (10–50 nm). In the case of samples with Fe and Co, larger oxide phases can be observed at the interface of the zeolite crystals, which can favor their agglomeration. In the inserted TEM images, the regular arrays of the zeolite Y pores are around 1 nm. After metal oxides’ immobilization, irregularly stretched lines indicating crystal imperfections of Ti-zeolite Y are not observed.

#### 2.1.3. SEM Analysis

**Figure 3:**
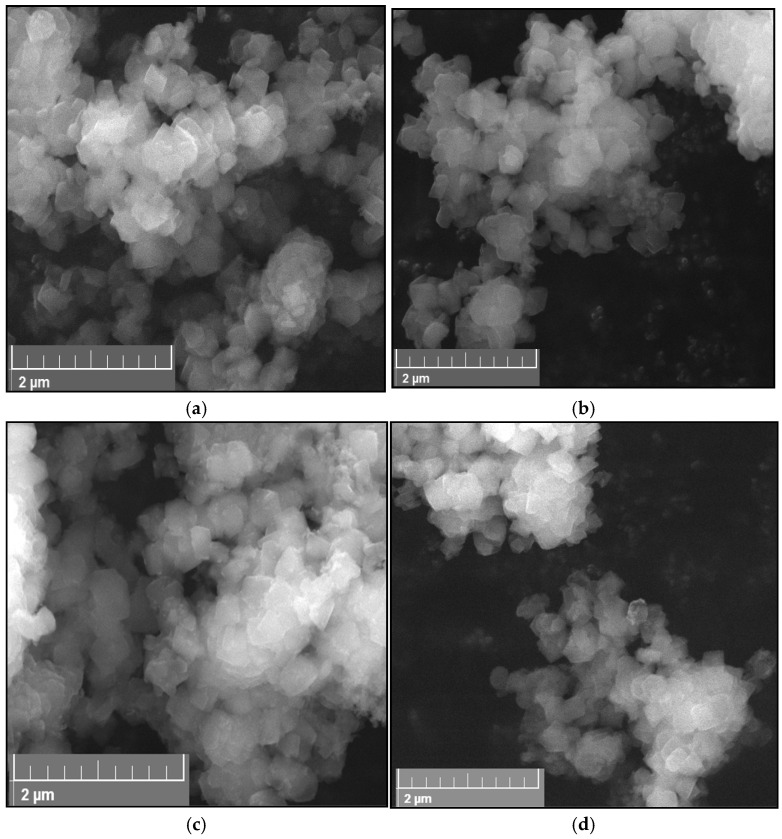
SEM images of (**a**) 1TY, (**b**) 1TYF, (**c**) 1TYC, and (**d**) 1TYN samples.

The morphology of synthesized materials was evidenced by scanning electron microscopy (SEM). SEM images of Ti-zeolite Y before and after immobilization of Fe, Co, and Ni oxides are presented in Figure 3. The typical morphology of zeolite Y (FAU zeolite), with octahedral crystals and multiple twinning polyhedrals [21,37], is evident in all the SEM images (Figure 3a–d). A slight change in morphology, possibly due to the crystals’ agglomeration, seems more visible for samples with supported iron and cobalt oxides (Figure S2). This confirms the effect of the oxide formations at the interface of the zeolite crystals, as evidenced in the TEM images (Figure 2b,c).

#### 2.1.4. N_2_ Physisorption

**Figure 4:**
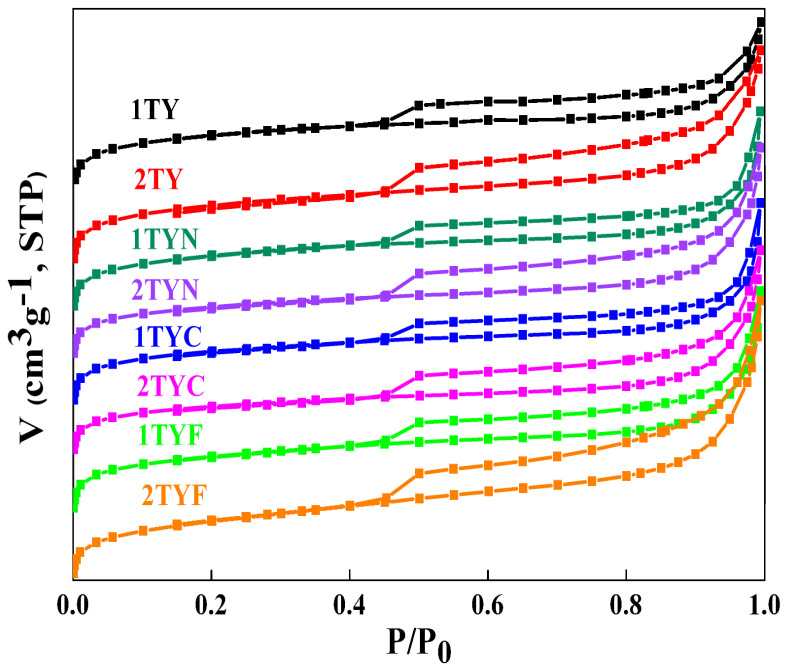
N_2_ adsorption–desorption isotherms of the investigated materials.

Figure 4 displays the N_2_ adsorption–desorption isotherms for all samples. Based on the IUPAC classification [38], all the isotherms are composites of type I (a) and type IV (a). This type of isotherm is characteristic of materials with dual porosity generated by micro and mesopores. The isotherms exhibit hysteresis loops at relative pressures higher than 0.45 and can be classified as type H4. The textural parameters of the samples are shown in Table 1.

As can be seen, the surface area of the samples impregnated with metal oxides decreased by 10 to 20% compared to the Ti-zeolite Y supports, but the percentage of micropores and mesopores remains almost constant, 88–90% (micropores) and 10–12% (mesopores). In the case of the 2TYF sample, the percentage of micropores is 84%, and that of mesopores is 16%. Regarding the total volume of the pores, it decreased in the samples impregnated with metal oxides compared to the support samples 1TY and 2TY. This indicates a uniform distribution of metal oxides on the internal surface of the pores. The exception is sample 2TYF, in which the percentage of micropores is 84% and that of mesopores is 16%. Regarding the total volume of the pores, the decrease in the samples impregnated with metal oxides compared to the support samples 1TY and 2TY is lower than in the case of the surface. It can also be seen that the decrease in total pore volume is between 1 and 13%, while the micropore volume decreases by a higher percentage, between 10 and 21%.

#### 2.1.5. Raman Spectroscopy

**Figure 5:**
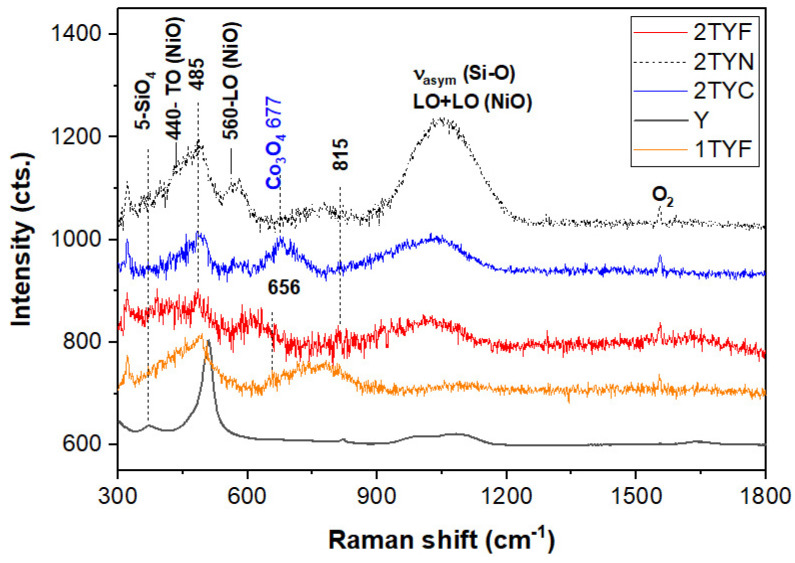
UV-Raman spectra of the (1/2)TY (F/C/N) samples and zeolite Y (*n* ≤ 6 and T stands for Al, Ti, and Si).

The UV Raman spectroscopy has been recently used for structural investigation of the surface of catalysts due to the limited penetration depth of the UV lasers [39]. Less intense and wider UV-Raman bands are present in the (1/2)TY(F/N/C) spectra in Figure 5 in contrast with the zeolite Y spectrum (372 cm^−1^, six-membered SiO_4_ rings, ~500 cm^−1^, four-membered SiO_4_ rings, 1006 cm^−1^, and 1081 cm^−1^, asymmetric stretching mode of the (Al/Si)-O linkages in tetrahedral coordination [40]). This is a consequence of the vitreous surface of the zeolite-supported catalysts.

The tinny band at about 672 cm^−1^ belongs to the A_1g_ modes of the Co_3_O_4_ [41] in the 2TYC spectrum. Moreover, the spinel structure, Co^2+^(Co^3+^)_2_O_2_^4−^, of Co_3_O_4_ was identified for the (1/2)TYC samples by Vis-Raman spectroscopy (Figure S3) at 194, 485, 521, 622, and 692 cm^−1^ (F_2g_, E_g_, F_2g_, F_2g_, and A_1g_). A two-phonon wide band at about 1060 cm^−1^ was reported for CoO [41]. However, the later band at 1050 cm^−1^ in Figure S3 can also belong to the T–O stretch, as pointed out in Table 2. Most of the NiO vibrations [42] (see Table 2) overlap those of SiO_2_ [43].

Given the low titania content and lack of the Raman bands of the anatase (398 cm^−1^, B_1g_, and 633 cm^−1^, E_g_) and/or rutile (611 cm^−1^ and 826 cm^−1^ due to A_1g_ and B_2g_ modes [44]) in Figure 5, tetrahedral framework titanium atoms were obtained in the (½)TY(F/N/C) powders by adding titanium acetylacetonate to the aluminosilicate gel, according to the synthesis method.

Given the strong UV adsorption due to the ligand-to-metal charge transfer transition of O^2−^-−Fe^3+^ → O^−^−Fe^2+^ was reported to occur below 300 nm [45], the Fe–O–Si bonds in a zeolite framework give only faint Raman signals regardless of the iron oxide content when an exciting laser line of 325 nm is used. Fan et al. [46] reported that Raman bands (collected with a 244 nm laser closed to the charge transfer transition) at about 516 and 1115 cm^−1^ in the Fe-ZSM-5 belong to the symmetric and asymmetric stretching modes of the Fe–O–Si bonds of the tetrahedral coordinated Fe^3+^. In the case of the 2TYF sample, the 1115 band might be obscured by the stronger Si–O stretching modes peaking up at 1027 cm^−1^ or extra-framework iron species (magnetite and/or hematite) that are present. The latter species should be confirmed by UV adsorption bands or shoulders at about 350 nm and 480 nm for hematite [45] (see below Section 2.3).

**Table 2:** Peak position, assignments, and references of the 2TY (N/C/F) and Y zeolite.

Y	2TYN	2TYC	2TYF	Assignment	Reference
372	396		391	Bending mode of double 6-membered SiO_2_ rings and E_g_ of Fe_2_O_3_ (412 cm^−1^)	[40,46]
	440	455		TO modes NiO, E_g_ of Co_3_O_4_ (470 cm^−1^) and n-membered SiO_4_ rings (*n* ≤ 6)	[41,43]
506	485	495	485	Breathing of the 4-membered SiO_2_ rings (4R, 508 cm^−1^) in 6-membered double rings (D6R, 490 cm^−1^) and Co_3_O_4_	[41,43]
	560			LO modes of NiO	[42]
	577			Al–O–Si stretching in connection with ring structures	[46]
			611	E_g_ modes of Fe_2_O_3_	[47]
			652	Symmetric Fe-O breathing A_1g_ modes	[45]
		677		A_1g_ of CoO_6_ (675 cm^−1^)	[41]
		705			
	740			2TO modes of NiO	[42]
	776			O–O stretching of adsorbed O_2_^2−^	[41]
1006				Asymmetric T–O stretch (T = Al and Si)	[40]
	1054	1032	1027	Asymmetric T–O stretch (T = Al, Ti, and Fe) and two-phonon modes of CoO	[41,43]
1081				Asymmetric T–O stretch (T = Al and Si)	[40]

### 2.2. Composition and Reduction in Photocatalysts

#### 2.2.1. XPS Spectroscopy

**Figure 6:**
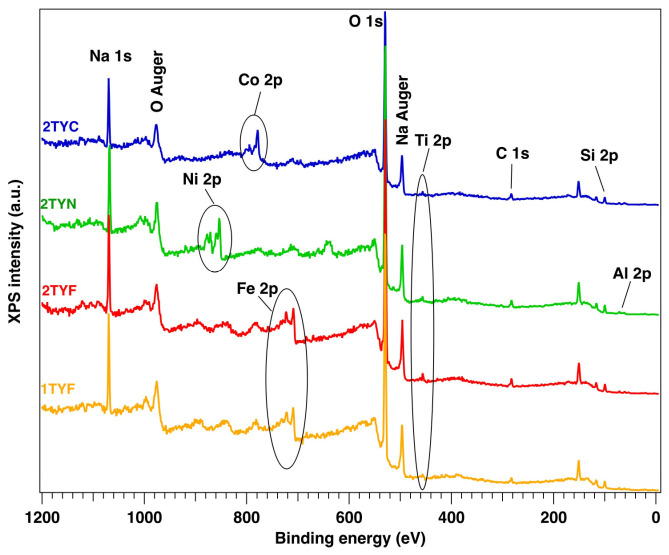
XPS full scan survey spectra for the samples, indicating all the elements.

**Figure 7:**
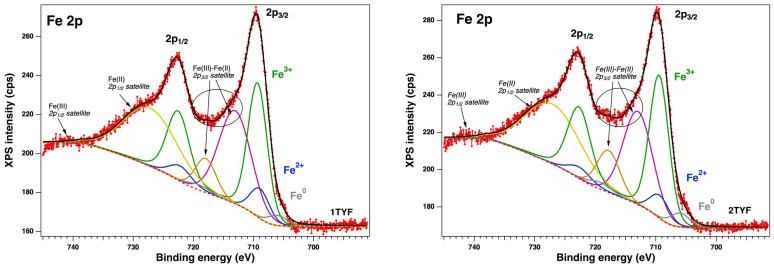
XPS spectra of Fe 2p (a) for the samples 1TYF and 2TYF and their deconvolutions with the interpretation. Red symbols—raw data, black line—fit, grey, blue, and green lines—the main components; the other components (yellow-green, orange and magenta lines) are the satellites of Fe(II) and Fe(III); the red dashed line is the Shirley background.

**Figure 8:**
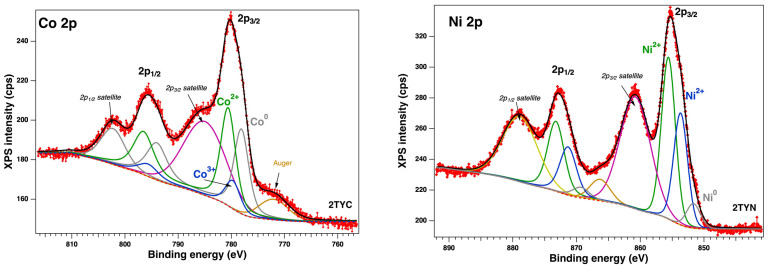
Fitted XPS spectra of Co 2p (red symbols—raw data, black line—fit, blue and green lines—the main components; the other components are the satellites and the Auger line of Co 2p) and of Ni 2p (red symbols—raw data, black line—fit, grey, blue and green lines—the main components; the other components (yellow-green, orange and magenta lines) are the satellites of Ni (2p); the red dashed line is the Shirley background.

The elemental composition and the chemical states of the sample surfaces were measured by X-ray photoelectron spectroscopy (XPS). The binding energies scale was calibrated to the standard value of C 1s, 284.6 eV. The core-level spectra were analyzed using Voigt profiles based on the methods described in ref. [48]. Thus, each component has its own background and could provide supplementary information [49,50]. The XPS survey (Figure 6) shows the following core levels: O 1s, Si 2p, Al 2p, Na 1s, C 1s, Ti 2p, Co 2p, Ni 2p, and Fe 2p. The analyzed fitted spectra of Fe 2p, Co 2p, and Ni 2p (Figure 7 and Figure 8) revealed the coexistence of different chemical states of each metal.

**Figure 9:**
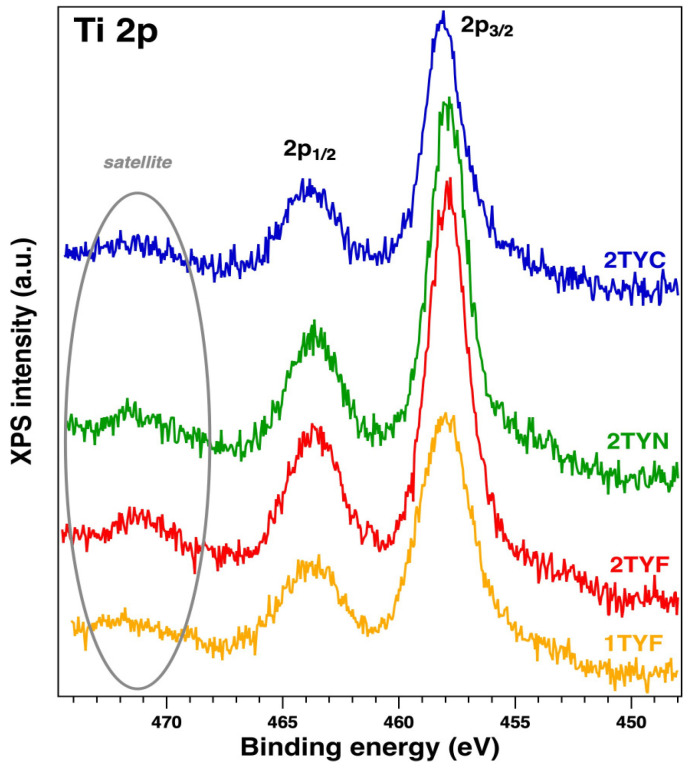
XPS spectra of Ti 2p for all the samples (mentioned on each spectrum).

Unfortunately, it is known that Fe 2p, Co 2p, and Ni 2p core-level spectra are complicated peaks due to the overlapping photoelectron lines, their satellites, and Auger lines in the spectrum. Thus, we extracted the surface chemical species by fitting the data with Voigt profiles [32] while using a Shirley background. The samples with Fe 2p showed up in three chemical states (Figure 7), according to the database: metallic (~707 eV), Fe^2+^ (~709 eV), and Fe^3+^ (~709.5 eV) [51]. Also, Co 2p presents (Figure 8) three main components and their satellites at binding energies (BE) of ~778.2 eV, 779.7 eV, and 780.5 eV, attributed to the metal, 3+, and 2+ chemical states [32,52,53] with a spin–orbit splitting of ~15.8 eV. The Ni 2p core level is illustrated in Figure 8, and the fitting revealed the coexistence of two chemical states: Ni^0^—851.7 eV (a small amount), Ni^2+^—853.7 eV (NiO), and 855.6 eV (Ni(OH)_2_), according to the tabulated data [54]. The spectra of Ti 2p for all samples show a main component at ~458 eV attributed to Ti^4+^ (see Figure 9). This attribution is also supported by the presence of the satellite at 471–472 eV [55]. Also, it can be seen from the different intensities of Ti 2p that this could be related to the interaction with the dopants.

The presence of titania in the synthesized materials was also confirmed by X-ray fluorescence. In the samples with lower titanium content, the percent of titania varied like this: 1.13% (1TYF), 1.11% (1TYC, 1TYN). For higher Ti content, the percent was 2.53 for 2TYF, 2.30 for 2TYC, and 2.54 for the 2TYN sample. The EDX results obtained for the first three samples are presented in Table S1. The dispersion of Fe, Co, and Ni oxides on Ti-containing zeolite Y crystals was evidenced by STEM microscopy (Figure S4). The images evidenced a good dispersion of the oxide nanoparticles on the surface of the support crystals.

#### 2.2.2. H_2_-Thermoprogrammed Reduction

The H_2_-TPR results illustrate a significant effect of metal oxides’ interaction with the support. Thus, Fe, Co, and Ni cations were immobilized as oxide species on zeolite Y support, with tetrahedral Ti^4+^ isolated into the network. Under these conditions, the oxide species of these cations in the mass concentration of 5% can disperse on the surface or locate inside the supercages of the zeolite Y.

**Figure 10:**
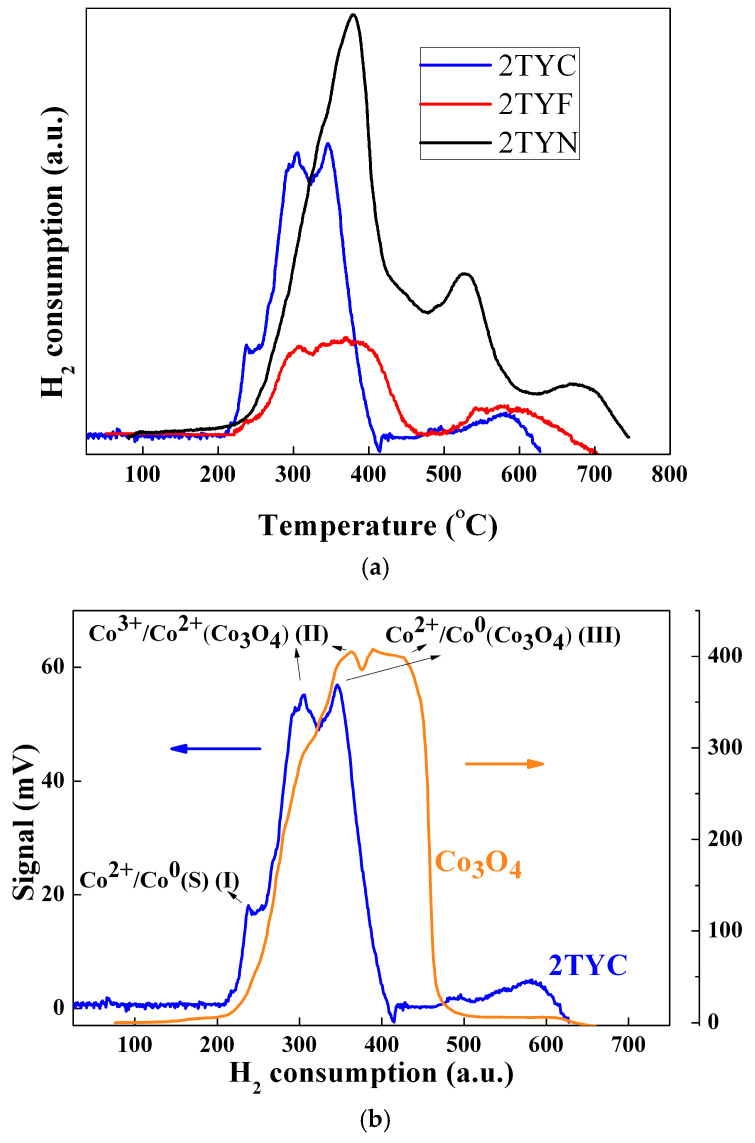
(**a**) H_2_-TPR profiles of samples with Fe, Co, and Ni oxides supported on zeolite support with 2% TiO_2_, and (**b**) H_2_-TPR comparative profiles of supported and commercial Co_3_O_4_ oxide.

The location of the oxide species influences the interaction with the support and, hence, their reduction temperature. Also, the interaction with Ti cations, especially in the case of the isolated oxides of these transition metals from channels or cages, influences the reduction in temperature. Figure 10 depicts H_2_-consumption profiles for samples obtained by immobilization of Fe, Co, and Ni on zeolite Y support with 2% TiO_2_. The reduction profiles obtained for the Ni/Ti-zeolite Y photocatalyst (Figure 10a) present one main peak with a maximum at ~378 °C and a weak shoulder at around 340 °C. The shoulder is due to the reduction of free NiO species, and the peak is associated with the reduction of NiO in weak interaction with the zeolite support [56,57]. The other two peaks of lower intensity, at ~524 °C and 667 °C, suggest the presence of different types of NiO species in stronger interaction with the zeolite support. The XPS results (Figure 8) confirm the presence of Ni^2+^ species on the zeolite surface for this sample.

The reduction profile for 2TYF material, obtained by immobilizing iron oxide species on zeolite Y with 2% TiO_2_, shows (Figure 10a) a little shoulder at 240 °C and two overlapping peaks with maximums at around 310 °C and 380 °C that can be connected to the conversion of Fe_2_O_3_ to Fe_3_O_4_ [58,59]. The first peak can be attributed to the reduction of the isolated Fe^3+^ species to Fe^2+^ and the second, broader one, to the oxide, which is probably located inside the supercages by different coordination with the framework oxygen atoms [60,61]. The last broad peak at around 600 °C can be related to the conversion of Fe_3_O_4_ to FeO. The iron oxide species that interact more or less strongly with the zeolite support justify this broad peak of the 2TYF sample reduction profile. The XPS spectra (Figure 7) of samples 1TYF and 2TYF indicate the presence of Fe^2+^, Fe^3+^, and also Fe^0^ on the surface. The fact that part of the iron species is already reduced to Fe^2+^ and their reduction to Fe^0^ can take place at higher temperatures (around 800 °C) justifies the reduced volume of hydrogen consumed compared to 2TYN (Figure 10a).

For the 2TYC sample containing supported cobalt oxides, reduction peaks were obtained at ~240 °C, 300 °C, 350 °C, and 580 °C. Compared to the pristine (Figure 10b), two reduction peaks were detected for pure Co_3_O_4_, at around 360 °C attributed to the reduction of Co^3+^ to Co^2+^ and at ~450 °C corresponding to the reduction of Co^2+^ to Co^0^ [62,63]. These peaks are shifted, for sample 2TYC, to lower temperatures (300 °C and 350 °C). Due to the dispersion on the support, both hydrogen adsorption and its interaction with the oxide species are favored. The XPS results (Figure 8) showed the presence of Co^2+^, Co^3+^, and Co^0^ on the surface of the 2TYC sample. The broader peak with a maximum at approximately 600 °C, only for supported cobalt oxide, may be due to a stronger interaction of Co^2+^ species with the support. In Figure 10, each H_2_-consumption profile shows a shoulder or a peak between 200 °C and 300 °C. Each shoulder is assignable to the adsorption on the surface of hydrogen. In the case of samples 2TYC and 2TYF, the peak and little shoulder at ca. 240 °C from the H_2_-consumption profiles can be due to the hydrogen spillover effect. For Fe_2_O_3_ supported on ZSM-5 zeolite, the effect of Fe^0^ and other transition metals on hydrogen atoms’ migration (H_2_ spillover) and the reduction of oxides were observed [61]. Thus, in the case of 2TYF and 2TYC samples, the spillover effect can be favored by Fe^0^ and Co^0^, as evidenced by XPS spectroscopy (Figure 7 and Figure 8), and by Ti^4+^ from the gel network of the zeolite Y support.

### 2.3. Optical Properties

#### 2.3.1. UV–Vis Spectroscopy

**Figure 11:**
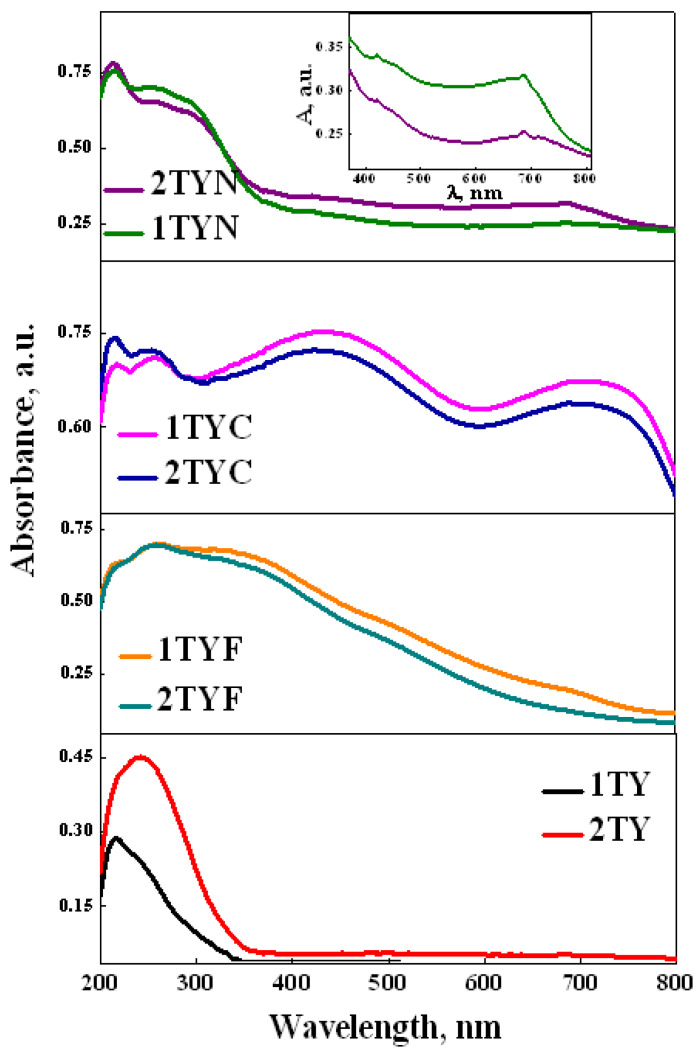
UV–Vis absorption spectra of the synthesized photocatalysts.

The optical properties of the synthesized materials were evaluated by UV–Vis absorption spectroscopy. The recorded spectra are presented in Figure 11 and suggest the successful incorporation of Ti in the aluminosilicate gel framework of zeolite Y. It is supported by the absorption band located at 210 nm, assigned to the ligand-to-metal charge transfer Ti^4+^O^2−^ → Ti^3+^O^−^ [64].

An additional broad absorption band appeared toward a higher wavelength (250 nm), was more visible for the samples with a higher Ti content (2%), and indicated the formation of extra-framework Ti species [64,65]. The small shoulder, located at 330 nm, is usually assigned to anatase traces. A strong redshift of absorption spectra was observed after iron oxide immobilization, given by the charge transfer of oxygen to octahedral Fe^3+^ from Fe_2_O_3_ nanoparticles. In addition, the band located at 350 nm suggests the formation of some extra-framework FeOx oligomers [66]. Also, Co-modified materials present two intense absorption bands in the visible range, with maximums located at 440 and 710 nm, assigned to the charge transfer transitions O^2−^ → Co^2+^ and O^2−^ → Co^3+^ [32,67]. Modification of Ti-zeolite Y supports with nickel oxide led to a slight broadening of the absorption band in the UV range due to O_2_ → Ni^2+^ charge transfer, which overlapped with the absorption bands given by the titanium species. Also, it was observed that two new broad absorption bands appeared in the visible range, located at 420 nm and 690 nm. These are related to the ^3^A_2g_ → ^3^T_1g_ (P) and ^3^A_2g_ → ^3^T_1g_ (F) transitions (Table 3) given by the presence of Ni^2+^ ions in octahedral or pseudo-octahedral symmetry [68,69].

**Figure 12:**
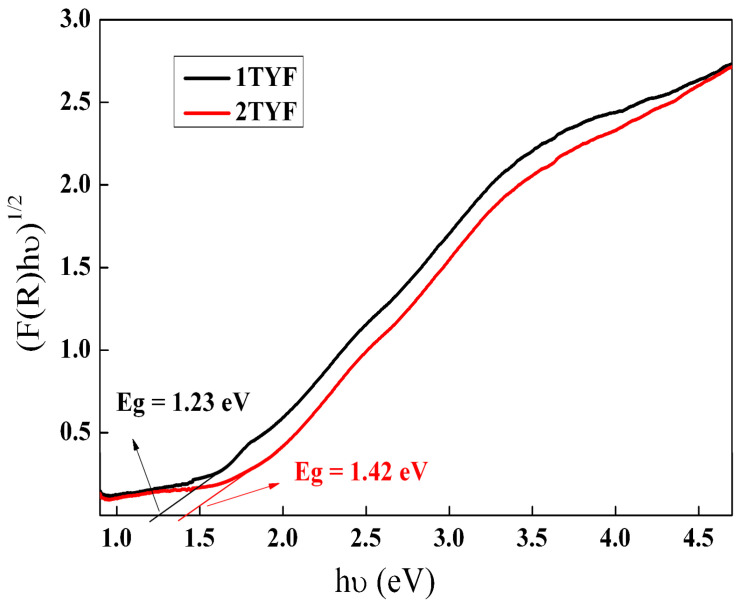

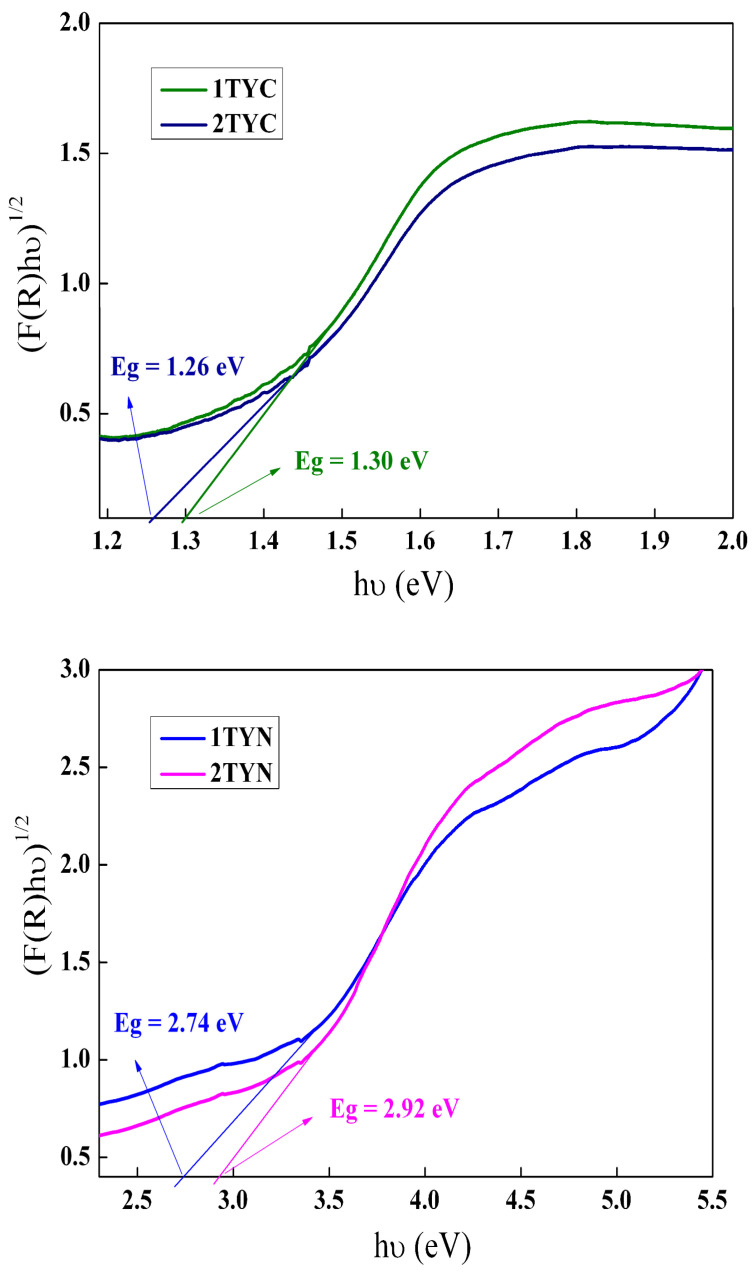
Band gap energy (Eg) determination for indirect transitions from the Tauc plot.

The band gap energies of all the samples were obtained from the Tauc plot using the Kubelka–Munk function, as in ref. [71], by extrapolating the linear part of the plot to the *x*-axis (Figure 12).

The band gap values determined for indirect transitions are summarized in Table 4 and showed a decrease compared with pristine TiO_2_ (3.2 eV [72]). An important narrowing of the band gap energy was observed by modifying the Ti-zeolite Y support with iron and cobalt oxide, obtaining values below 1.5 eV (Table 4). It means that less energy is required for e^−^ transfer, improving the photocatalytic process. Although the immobilization of nickel oxide also leads to a decrease in the energy of the band gap, the effect is not as important as in the case of iron and cobalt oxides.

**Figure 13:**
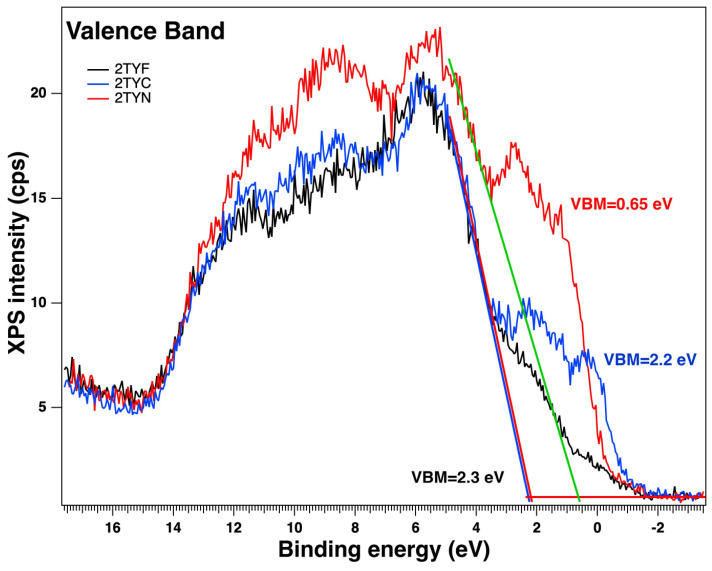
Valence band XPS spectra of the photocatalyst, with linear fits through the highest energy slopes of the valence band regions.

In order to represent the reaction mechanisms of the synthesized materials, the values of their valence band (VB) energy for the photocatalysts were obtained from the valence band (VB) XPS measurements (Figure 13).

The XPS valence band spectra revealed the VB maxima of 2.3 eV for the 2TYF sample, very close to the VB potential of 2TYC (2.2 eV) and 0.65 eV for 2TYN. Further, the conduction band (CB) minima of the samples were calculated using the following Equation (1):

E_CB_ (vs. NHE) = E_VB_ (vs. NHE) − E_g_, (1)

where E_CB_ is CB potential, E_VB_ is VB potential, and E_g_ is band gap energy [73]. So, the obtained CB potentials for the samples were 0.88 eV for 2TYF, 0.94 eV for 2TYC, and −2.27 eV for 2TYN.

#### 2.3.2. Photoluminescence Spectroscopy

**Figure 14:**
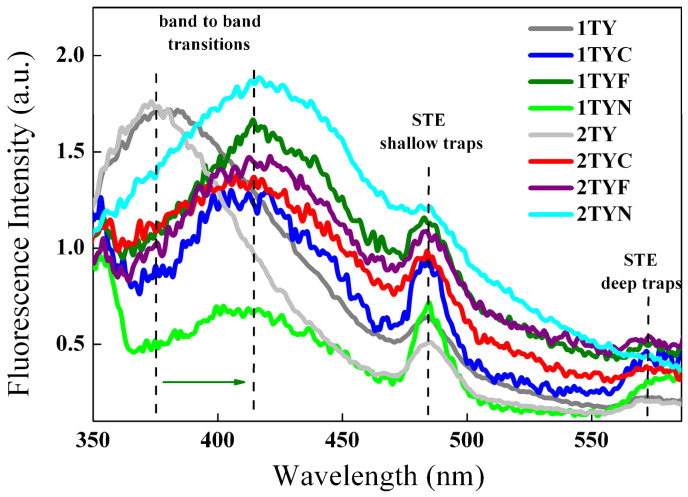
PL spectra of Ti-zeolite Y and Fe/Co/Ni modified Ti-zeolite Y with different TiO_2_ content (λ_exc._ = 320 nm), green arrow suggests the shift of the signals attributed to the band to band transitions after modification with Fe, Co and Ni.

The photoluminescence (PL) technique has been used to investigate the effect of 3d metals from the VIII group on the optical and photochemical properties of Ti-zeolite Y. The recorded PL spectra of Ti-zeolite Y and Fe/Co/Ni-modified Ti-zeolite Y, using an excitation wavelength of 320 nm, are shown in Figure 14. Ti-zeolite Y materials with different amounts of TiO_2_ display two clear PL emissions peaks related to the indirect band-to-band recombination of photogenerated electron–hole pairs (in the high energy range) [74] and to the self-trapped excitons (STE) formed by electrons binding to the oxygen vacancies and defects, respectively (in the low energy range) [70,74,75].

Modification of aluminosilicate gel containing titanium in the network with oxides of 3d metals from the VIII group leads to obtaining three obvious PL signals at about 415, 485, and 575 nm, respectively. It is reported that the bands appearing in the visible range are mainly related to surface defects, band edge-free excitons, and oxygen vacancies [76,77,78]. The type of dopant species like Fe, Co, or Ni seems to have an important effect on the photoluminescence properties due to the different effects on the separation of photogenerated electron(e^−^)/holes(h^+^) pairs and their recombination process. Figure 14 shows that the post-synthesis modification of aluminosilicate gel containing titanium in the network with cations of 3d metals from the VIII group led to a shift in the lower energy range and to a quenching of the first PL emission signal. This is most probably due to the formation of some sub-bands (surface states) by the addition of 3d metallic species, so the transfer of excited electrons from the bottom of the conduction band to the top of the valence band takes place via new sub-bands through a non-radiative process firstly and a radiative one, secondly [79]. The formation of new sub-bands leads to a delay in the electron–hole repair, which is desirable in photocatalysis. The second PL signal seems to keep its position but increases in intensity after the immobilization of 3d metallic oxides. It is related to the formation of new oxygen vacancies and defects as shallow trap centers, iron oxide having the most pronounced effect, followed by cobalt oxide. Also, the results showed a new broad PL band appearing in the lower energy domain, assigned to deep defects and oxygen vacancies [70,75].

### 2.4. Photocatalytic Activity

#### 2.4.1. Photocatalytic Degradation of Cefuroxime

**Figure 15:**
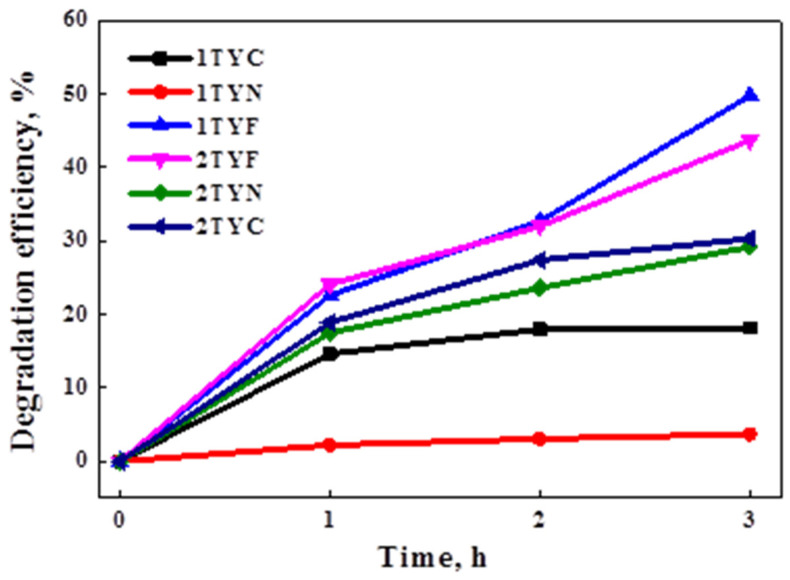
Photocatalytic performances of the samples under visible light irradiation.

The photocatalytic performances of the samples were evaluated in the degradation of cefuroxime (β-lactam) antibiotic from water under visible light irradiation. The results are shown in Figure 15 and indicate the highest degradation efficiencies for the samples modified with iron oxide. This is explained by the lower value of the band gap energy (1.23 eV for 1TYF and 1.30 eV for 2TYF, Table 4), thus contributing to the electron transfer process under visible light irradiation.

Noteworthy, the photocatalytic activity of Ti-aluminosilicate gel modified with transition metals from the VIII group (Fe, Co, and Ni) exhibits a direct relationship with the PL signals associated with surface trap excitons processes. It suggests the important effect of oxygen vacancies and defects obtained during the synthesis, which bind the photogenerated electrons, preventing e^−^/h^+^ pairing and thus improving their availability to further interact with oxygen molecules adsorbed on the surface and generate superoxide radicals, active in the degradation process.

Better photocatalytic performances were obtained for the photocatalysts reported in this work compared to similar materials previously obtained by our group through TiO_2_ immobilization on zeolite Y by the impregnation method [32]. Comparing the results, a significant decrease in photocatalytic activity can be observed for samples with incorporated Ti in the zeolite Y network and immobilized nickel oxide. The physicochemical properties of materials that are strongly influenced by the synthesis method, the nature of the pollutant, and the experimental test conditions determine their photocatalytic performances. Fe-, Co-, or Ni-TiO_2_ nanocomposites have been used in different reactions for the photocatalytic degradation of several pollutants from wastewater, such as Acid Orange 7 and Methylene Blue, with high photocatalytic efficiencies [80,81], but similar materials with Ti dispersed in the zeolite framework by direct synthesis have not yet been reported, to our knowledge.

**Figure 16:**
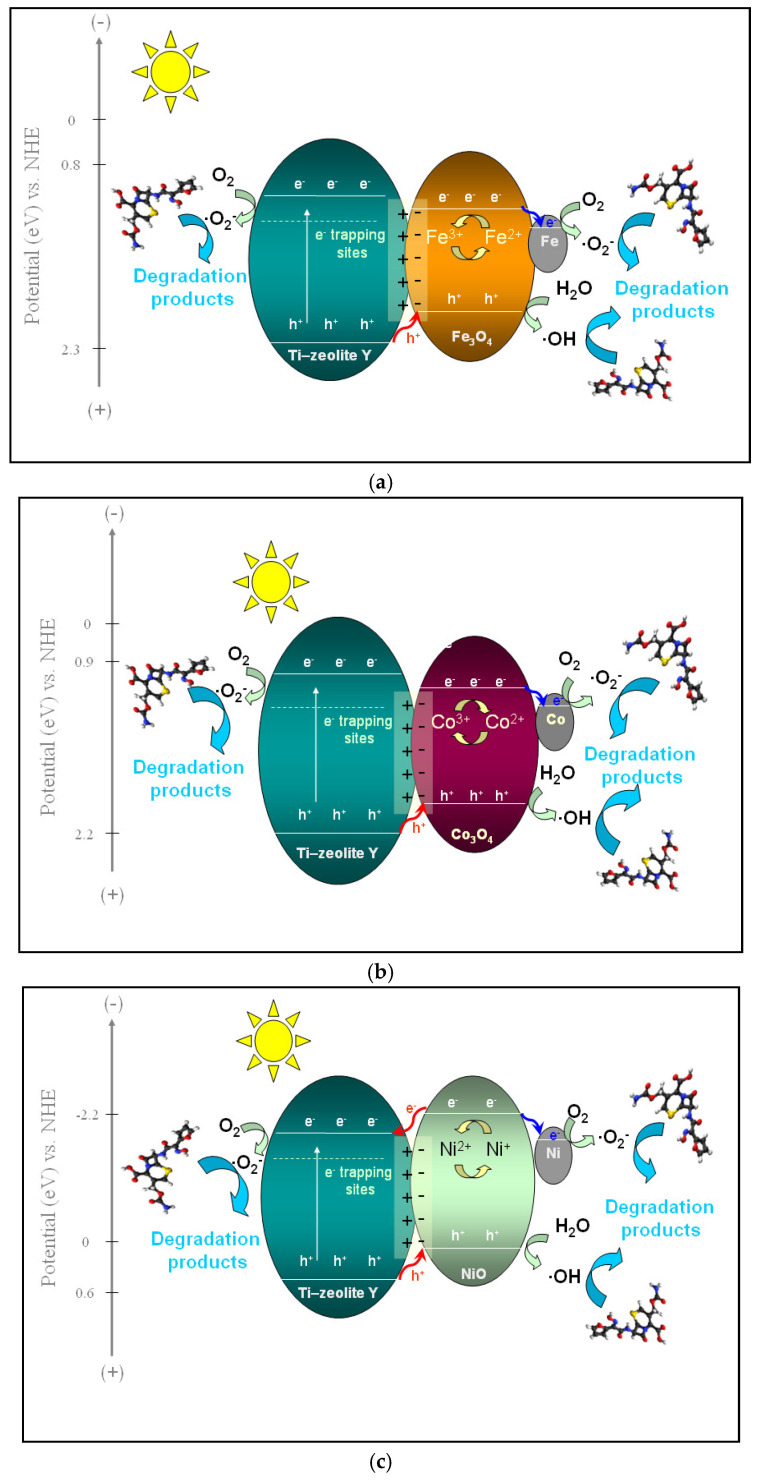
Possible mechanism of photocatalytic degradation of cefuroxime over Ti-zeolite Y modified with (**a**) Fe, (**b**) Co, or (**c**) Ni oxides.

It was shown that in the process of TS-1 doping with Fe or Ni, the migration of electrons is from iron species to the support, respectively, from the support to nickel species [82]. The different interactions with the support and different energies of valence and conduction bands of the photocatalysts with Fe and Ni compared to Ti-containing zeolite support influence the mechanism of the photocatalytic reaction as well as the degradation efficiency (Figure 15). The proposed mechanisms of cefuroxime degradation over the prepared photocatalysts are presented in Figure 16.

Adding various p-type semiconductors such as Fe_3_O_4_, Co_3_O_4,_ and NiO to Ti-containing zeolite Y (n-type semiconductor) with titanium incorporated in the network leads to the formation of a p–n type heterojunction. There is an internal electric field that ensures the spatial separation of the photogenerated charge carriers and directs the transfer of holes from an n-type semiconductor to a p-type semiconductor and the transfer of electrons in reverse [83]. According to the band edge position, a difference appears between the three types of synthesized materials. As can be seen in Figure 16, while the holes generated in the valence band (VB) of TiO_2_ are transferred to the VB of all the used p-type semiconductors, the electron transfer from the conduction band (CB) of p-type semiconductors to the CB of TiO_2_ is allowed only for NiO. However, there is a transfer of electrons from the conduction band of the 3d metal oxides (Fe_3_O_4_, Co_3_O_4_, and NiO) to the metallic species formed, as was suggested by the XPS data (Figure 7 and Figure 8) for each of the three types of photocatalytic systems. Thus, the recombination of e^−^/h^+^ pairs is reduced.

Meanwhile, the high dispersion of iron oxide, as was suggested by XRD results, ensures an efficient p–n heterojunction and, hence, better separation of photogenerated charge, improving the photocatalytic performances. Under light irradiation, the separated electrons and holes are available to interact with O_2_ and H_2_O from the reaction medium, leading to the generation of different reactive species that are free to degrade the cefuroxime molecules. As illustrated in Figure 16, the photogenerated electrons reduce O_2,_ producing superoxide (·O_2_^−^) radicals, while the separated holes oxidize H_2_O molecules, forming hydroxyl (·OH) radicals in the system. These two reactive oxygen species thus obtained are considered to be the main ones involved in the photocatalytic degradation of organic pollutants in water [73,80,84]. To further investigate the degradation mechanism and to clarify the role of each reactive species, scavenger experiments were carried out.

#### 2.4.2. Scavenger Experiments

Exploration of the degradation mechanism depending on the type of 3d metal oxide supported on Ti-incorporated zeolite Y was performed using different quenching molecules, which interact specifically with the main reactive species resulting under irradiation.

**Figure 17:**
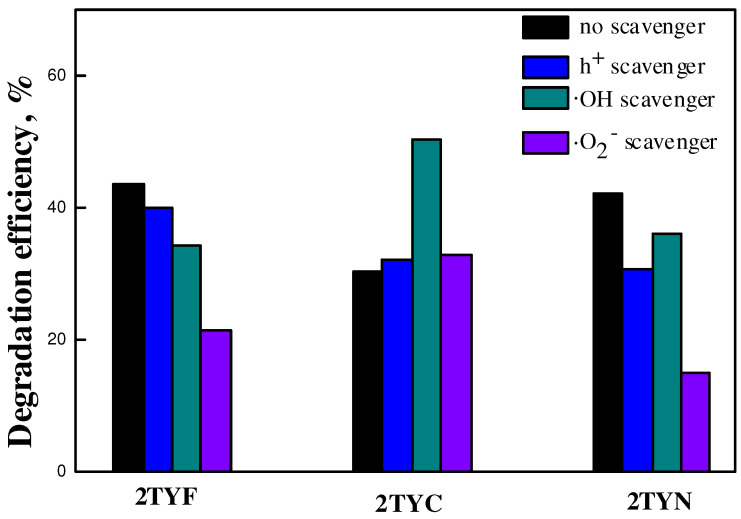
Scavenger experiments for the photocatalytic degradation of cefuroxime using Ti-zeolite Y modified with Fe, Co, or Ni oxides.

As shown in Figure 17, the scavenger addition in the reaction system led to a distinct decrease in the efficiency of cefuroxime degradation, indicating that several reactive species are involved in the process. The addition of the h^+^ scavenger had a moderately decreasing effect on the photocatalytic efficiency, indicating that the photogenerated holes also participated in the degradation process. For all the samples, it was observed that the addition of p-BQ significantly decreased the degradation reaction, suggesting that superoxide radicals (·O_2_^−^) played a key role in the oxidation of cefuroxime.

A considerable decrease in the photocatalytic efficiency by adding ·O_2_^−^ scavenger was found in the case of the sample modified with Ni oxide, suggesting that for this material, the degradation of cefuroxime takes place mainly through superoxide species. This behavior can be explained by the higher probability of superoxide radical formation, led by a better separation of electron–hole pairs. The significant increase in efficiency for the 2TYC photocatalyst in the presence of the ·OH scavenger evidenced the effect of 3d metal oxides and their interaction with Ti-incorporated into the zeolite Y gel network on quenching molecule degradation.

#### 2.4.3. Stability of the Photocatalysts

**Figure 18:**
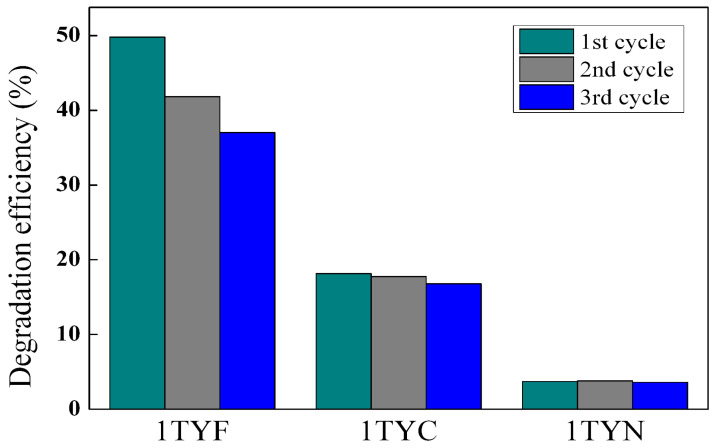
Photocatalytic performances of the photocatalysts during three consecutive recycling reactions.

The synthesized materials show good stability after three consecutive cyclic reactions, as shown in Figure 18. Only for the 1TYF sample was there a tendency to decrease the photocatalytic efficiency.

**Figure 19:**
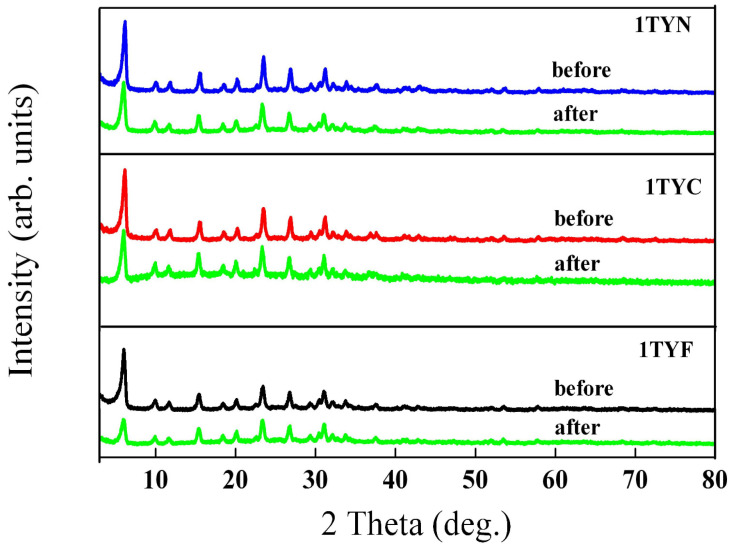
Wide-angle X-ray diffraction patterns of the samples before and after three consecutive recycling experiments.

The X-ray diffractograms (Figure 19) of the used photocatalysts indicated a slight decrease in the peak intensity for samples with Co and Ni. In the case of the sample with Fe, the variation in intensity after the three consecutive reaction cycles is more significant and is correlated with the decrease in the crystalline phase percent in the sample. This may be the result of the interaction of iron species with the support, which reduces its stability. The possibility of Fe^3+^ incorporation as a tetrahedral coordinated ion in both the zeolite framework and the extra framework was confirmed by Raman (Table 2) and EPR spectroscopy [20]. SEM images of the used samples (Figure S5) do not indicate changes in morphology. The less contoured forms of the crystalline assemblies observed for the 1TYF sample confirm the XRD results. Therefore, the stability of the samples decreases with their activity.

## 3. Conclusions

In the present work, an aluminosilicate gel was used as a raw material for the synthesis of efficient photocatalysts for the degradation of β-lactam antibiotics. Titanium was incorporated in different concentrations (1 and 2%) into the zeolite network by modifying the gel during the zeolitization process. A series of 3d metals from the VIII group, such as Fe, Co, and Ni, were supported on Ti-containing zeolite Y, and their photocatalytic properties were studied. H_2_-TPR, Raman, XPS, and UV–Vis spectroscopy results showed the formation of Fe_3_O_4_, Co_3_O_4_, and NiO, along with small amounts of metals for all obtained samples. These species and their interaction with support facilitated the charge separation across the p–n heterojunction interface and also the presence of oxygen vacancies and surface defects, which can bind the photogenerated electrons. The influence of metallic species that receive electrons from the conduction band of the corresponding oxide on the photocatalytic reaction mechanism and the effect of each metal oxide on degradation efficiency were also evidenced. The best results were obtained for iron-supported samples. In this case, a stronger interaction of the iron with the support was observed, which can explain both the higher activity and the lower stability of the photocatalysts.

## 4. Materials and Methods

### 4.1. Materials

#### 4.1.1. Chemicals

For the synthesis of titanium-zeolite Y supports were used in sodium silicate solution (26.5 wt. % SiO_2_, 10.6 wt. % Na_2_O), sodium aluminate (NaAlO_2_), and titanium acetylacetonate ((CH_3_)_2_CHO]_2_Ti(C_5_H_7_O_2_). The basic medium required for the zeolitization process was ensured by using NaOH (98 wt. %). All the chemicals were purchased from Sigma-Aldrich (St. Louis, MO, USA). The post-synthesis modification of the supports was made by using iron (III) nitrate nonahydrate (Fe(NO_3_)_3_·9H_2_O (Sigma Aldrich), nickel (II) nitrate (Ni(NO_3_)_2_·6H_2_O), and cobalt (II) nitrate (Co(NO_3_)_2_·6H_2_O) from Merck (Darmstadt, Germany).

For the photocatalytic experiments, cefuroxime (C_16_H_16_N_4_O_8_S) was used, and for the scavenger experiments, potassium iodide (KI), ethanol (C_2_H_5_OH), and p-benzoquinone (C_6_H_4_O_2_). All of these chemicals were purchased from Merck (Darmstadt, Germany).

#### 4.1.2. Sample Preparation

Titanium-containing Y zeolite materials with different titanium concentrations (noted xTY, x = 1, 2 for 1% and 2% calculated titania content, respectively) were obtained by a seed-assisted method. This method ensures the incorporation of titanium into the aluminosilicate gel network corresponding to zeolite Y by direct synthesis, using titanium acetylacetonate as a titanium precursor. The two sol–gels prepared as has been described before by our group [21] were hydrothermally treated to obtain the crystalline structure specific to faujasite Y. After filtration, the precipitates were washed with deionized water, dried at 60 °C, and calcined at 600 °C.

**Figure 20:**
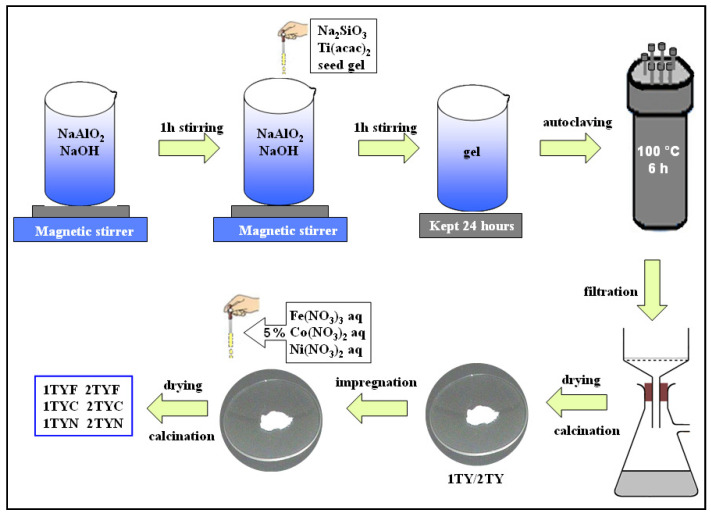
Flowchart for the synthesis of the photocatalysts.

The two supports thus obtained were further modified with 5 wt. % 3d metal oxide by the impregnation method using an aqueous solution of Fe(NO_3_)_3_, Co(NO_3_)_2,_ or Ni(NO_3_)_2_. The amount of iron, cobalt, and nickel precursor was equivalent to 5 wt.% of oxide in each sample and was dispersed in a calculated volume of water, according to the impregnation capacity of the support (impregnation ratio aqua:powder was 1 for 1TY and 0.93 for 2TY). The samples were firstly dried at room temperature overnight and then at 100 °C for 8 h. Co and Ni-modified photocatalysts were calcined at 450 °C for 6 h and Fe-modified samples at 500 °C for 5 h in air with a heating rate of 2 °C/min. The obtained materials were noted as xTYMe, where x is 1 or 2 and represents the concentration of titanium species, and Me is the 3d metal used for impregnation (Fe, Co, or Ni). A schematic representation of the photocatalysts’ preparation process is presented in Figure 20.

### 4.2. Methods of Characterization

X-ray diffraction (XRD) patterns were recorded using a Rigaku Ultima IV diffractometer (Rigaku Corp., Tokyo, Japan) with Cu Kα (λ = 0.15406 nm) within a 2θ range ranging from 5° to 80° with a speed of 2°/min and a step size of 0.02°. Phase identification was performed using Rigaku’s PDXL software (Version 1.8) connected to the ICDD database.

Elemental analysis of the samples was performed in a vacuum using a Rigaku ZSX Primus II spectrometer (Tokyo, Japan). The results were analyzed using EZ-scan combined with Rigaku SQX fundamental parameters software (Version 5.18) (standard less), which is capable of automatically correcting all matrix effects, including line overlaps. Structure and morphology were evidenced by transition electron microscopy using a JEOL 2100 TEM microscope (Tokyo, Japan) and scanning electron microscopy using a TESCAN Lyra3 XMU SEM microscope (Brno, Czech Republic). SEM was operated at 20 kV and a 15 mm working distance. Images have been obtained using secondary electron (SE) mode. TEM was operated at 200 kV. Images have been obtained in bright field mode.

The textural characterization of the samples was performed by N_2_ physisorption analysis using a Micromeritics ASAP 2020 instrument (Norcross, GA, USA). The samples were degassed under vacuum for five hours at 300 °C before each measurement. The BET model was used to calculate the apparent surface areas from the adsorption branches, while the amount of nitrogen adsorbed at the relative pressure of 0.99 was used to calculate the total pore volume. The micropore and mesopore volumes, together with the micropore and mesopore surface areas, were calculated using the t-plot method.

The XPS spectra were obtained using an AXIS Ultra DLD (Kratos Surface Analysis, Manchester, UK) setup, using Al Kα1 (1486.74 eV) radiation produced by a monochromatized X-ray source at an operating power of 144 W (12 kV × 12 mA). The survey spectra were recorded using hybrid lens mode with a 160 eV pass energy and slot aperture, and the high-resolution spectra were measured with a 40 eV pass energy.

Hydrogen temperature-programmed reduction (H_2_-TPR) of the samples was performed by means of a ChemBET 3000-Quantachrome (Pittsburgh, PA, USA) with a thermal conductivity detector (TCD). A continuous flow of 5 vol% H_2_ in Ar (70 mL/min) over 50 mg of photocatalyst was used, with a heating rate of 10 °C/min.

The UV-Raman spectra of the xTY(F/N/C) samples were recorded using a LabRam HR800 spectrometer (Horiba France SAS, Palaiseau, France) equipped with a 325 nm laser line. Vis-Raman spectra of the (½)TYC samples were collected by means of a Jobin -Yvon T64000 spectrometer (Horiba Jobin-Yvon, Palaiseau, France). The samples were analyzed with a wavelength of 532 nm and a power of 0.5 mW.

DR-UV/Vis spectroscopy was used to detect the coordination states of 3d metallic species added to the zeolite Y framework during the synthesis (in the case of Ti) or post-synthesis by the impregnation method (for Fe, Co, and Ni). The spectra of the samples were recorded by means of a JASCO V570 spectrophotometer (Tokyo, Japan) in the range of 200–1000 nm.

The photoluminescence spectra of the powders were recorded by means of an FLSP 920 spectrofluorimeter (Edinburgh Instruments, Livingston, UK) with a Xe lamp as an excitation source (λ_exc_ = 320 nm). For all measurements, the excitation and emission slits were 7 nm.

The photocatalytic tests were conducted under stirring in a closed room at 30 °C by adding 10 mL aqueous solution of cefuroxime, CFX (5 mg/L), and 20 mg of the photocatalyst. The reaction mixture was stirred in darkness for 30 min to allow the adsorption of cefuroxime molecules on the hydrophobic surface of zeolite materials. Further, a halogen lamp (2 × 60 W) with a filter for visible light was used for irradiation. At intervals of one hour, a fixed volume of the mixture was taken out, and the photocatalyst was separated using a Millipore syringe filter of 0.45 μm. The filtered solution was spectrophotometrically measured using the same JASCO V570 UV–Vis spectrophotometer, reading the maximum absorbance of CFX at λ = 280 nm. The photocatalytic degradation efficiency was obtained using the relation (A_0_ − A_t_)/A_0_ × 100, where A is the absorbance of the solution at time t = 1, 2, or 3 h and A_0_ is the initial absorbance of cefuroxime at t = 0. For scavenger experiments, 0.1 mmol of potassium iodide, ethanol, and p-benzoquinone were added to the CFX solution as h^+^, ·OH, and ·O_2_^−^ scavengers, respectively. The procedure was similar to the photocatalytic experiments. The results were presented for the case of irradiation for 3 h.

## Figures and Tables

**Table 1:** Textural parameters of the investigated materials.

Sample	BET Surface Area (m^2^/g)	Micropore Area (m^2^/g)	Mesopore Area (m^2^/g)	Total Pore Volume (cm^3^/g)	Micropore Volume (cm^3^/g)
1TY	649	585	64	0.316	0.221
2TY	591	521	70	0.320	0197
1TYC	588	532	56	0.314	0.201
2TYC	481	433	48	0.278	0.163
1TYF	573	510	63	0.319	0.193
2TYF	489	409	80	0.322	0.156
1TYN	551	484	67	0.298	0.183
2TYN	517	461	56	0.293	0.174

**Table 3:** Assignments of the absorption peaks recorded for the samples.

Sample	Absorption Peak Position	Assignment	Ref.
1TY 2TY	210 nm	framework Ti species (Ti^4+^O^2−^ → Ti^3+^O^−^ charge transfer)	[64]
	250 nm	extra-framework Ti species	
	330 nm	anatase traces	
1TYF 2TYF	260 nm	charge transfer of oxygen to Fe^3+^ cations in octahedral coordination	[66]
	350 nm	extra-framework FeO_x_ oligomers	
	480 nm	oxygen-to-metal charge transfer transitions that involve octahedral Fe^3+^ species	
1TYC 2TYC	440 nm	O^2−^ → Co^2+^ charge transfer transition	[32,67]
	710 nm	O^2−^ → Co^3+^ charge transfer transition	
1TYN 2TYN	420 nm	^3^A_2g_ → ^3^T_1g_ (P) charge transfer transition	[70]
	690 nm	^3^A_2g_ → ^3^T_1g_ (F) charge transfer transition	

**Table 4:** Band gap values of the photocatalysts.

Sample	1TYF	1TYN	1TYC	2TYF	2TYN	2TYC
Eg (eV)	1.23	2.74	1.30	1.42	2.92	1.26

## Data Availability

The data presented in this study are openly available in the article.

## Supplementary Materials

The following supporting information can be downloaded at: https://www.mdpi.com/article/10.3390/gels10020129/s1, Figure S1: XRD patterns of Ti-zeolite Y supports; Figure S2: SEM images of (a) 1TY, (b) 1TYF, (c) 1TYC, and (d) 1TYN samples; Figure S3: Vis-Raman spectra of the (½)TYC materials; Figure S4: STEM images of the samples with Fe, Co, Ni supported on Ti-containing zeolite Y; Figure S5: SEM images of the samples before and after photocatalytic reactions; Table S1: EDX results.
