# Supplementary material for: Composite Photocatalysts with Fe, Co, and Ni Oxides on Supports with Tetracoordinated Ti Embedded into Aluminosilicate Gel during Zeolite Y Synthesis
Source: Gels. 2024 Feb 5;10(2):129. doi: 10.3390/gels10020129 (PMC10888282; doi:10.3390/gels10020129)
Supplement: Supplementary file 1 [file gels-10-00129-s001.zip › gels-2746580-supplementary.pdf]

gels

Supplementary data

Comparative Effect of Fe, Co, and Ni Oxides Supported on Ti-Incorporated Zeolite Y on Photocatalytic Performances

Gabriela Petcu[1,*], Elena M. Anghel[1], Irina Atkinson[1], Daniela C. Culita[1], Nicoleta G. Apostol[2], Andrei Kuncser[2], Florica Papa[1], Adriana Baran[1], Jean-Luc Blin[3] and Viorica Parvulescu[1,*]

[1] Institute of Physical Chemistry "Ilie Murgulescu" of the Romanian Academy, 202 Splaiul Independentei, 060021 Bucharest, Romania; gpetcu@icf.ro (G.P.), manghel@icf.ro (E.M.A.), iatkinson@icf.ro (I.A.), dculita@icf.ro (D.C.C.), floricapapa@gmail.com (F.P.), adibaran@gmail.com (A.B.), vpirvulescu@icf.ro (V.P.)

[2] National Institute of Materials Physics, Atomistilor 405A, 077125 Magurele, Romania; nicoleta.apostol@infim.ro; andrei.kuncser@infim.ro

[3] Faculty of Sciences and Technology, University of Lorraine, CNRS, L2CM, F-54000 Nancy, France; jean-luc.blin@univ-lorraine.fr

* Correspondence: gpetcu@icf.ro (G.P.), vpirvulescu@icf.ro (V.P.)

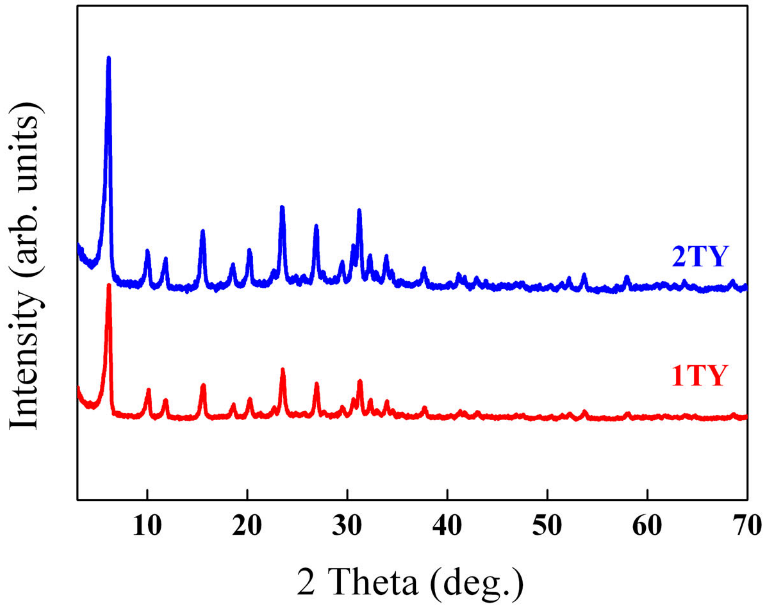

Figure S1. XRD patterns of Ti-zeolite Y supports.

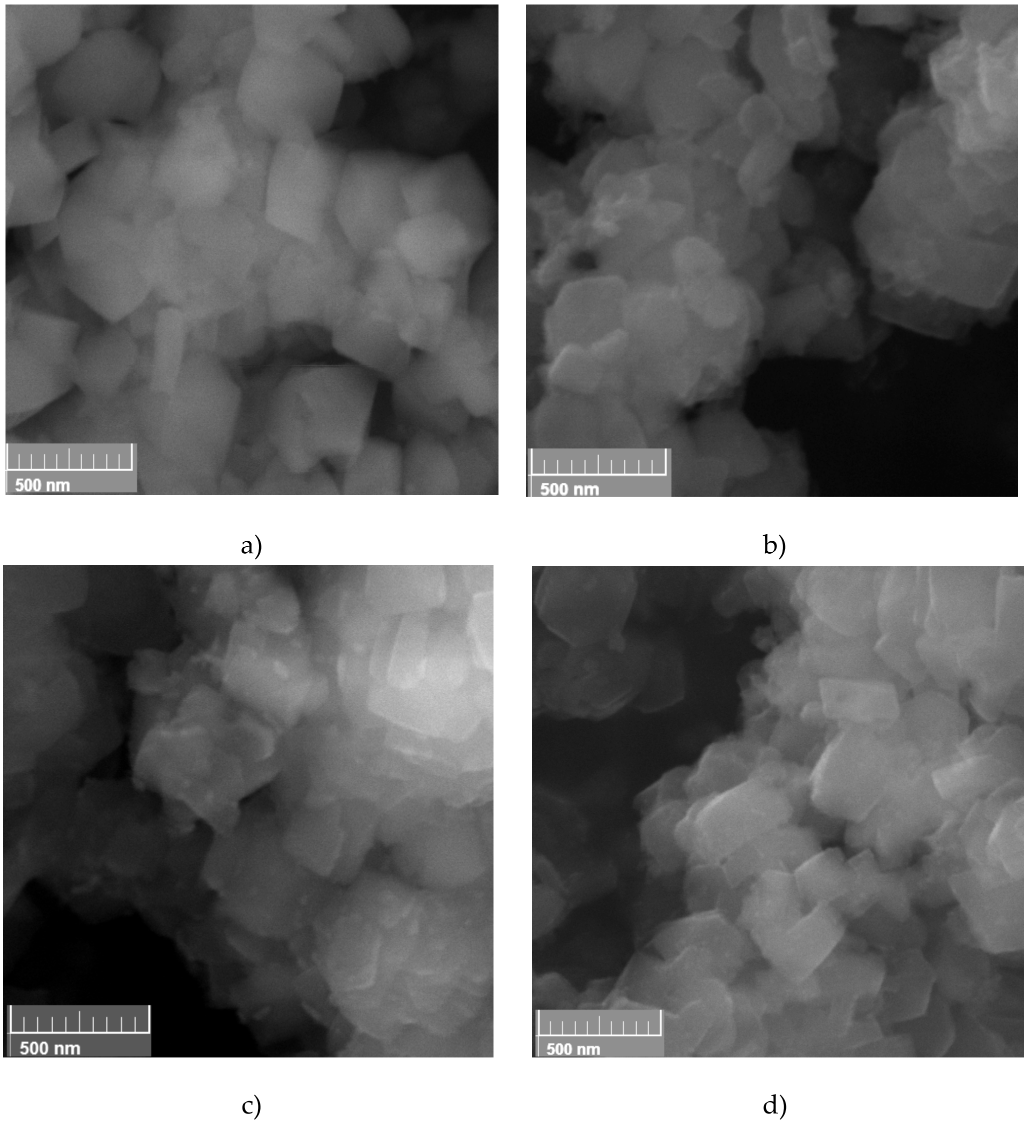

Figure S2. SEM images of a) 1TY, b) 1TYF, c) 1TYC, and d) 1TYN samples

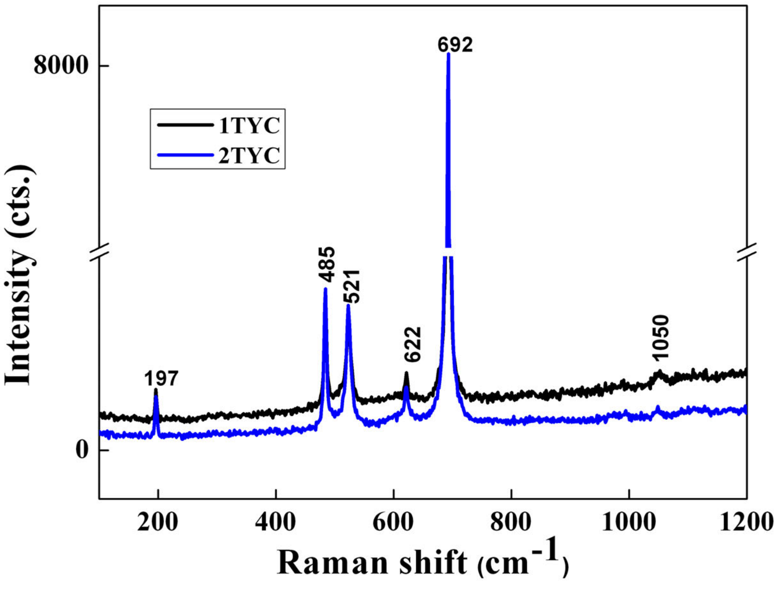

Figure S3. Vis-Raman spectra of the (½)TYC materials.

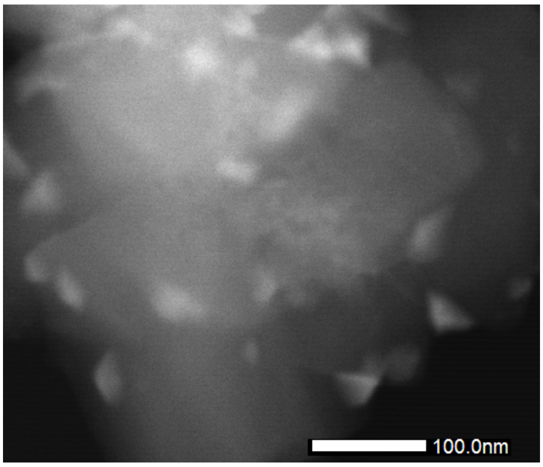

1TYF

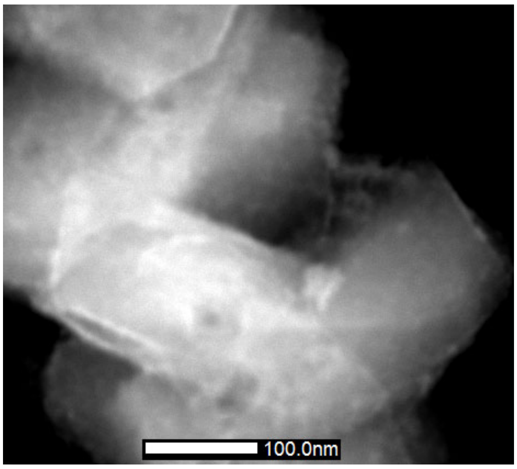

1TYC

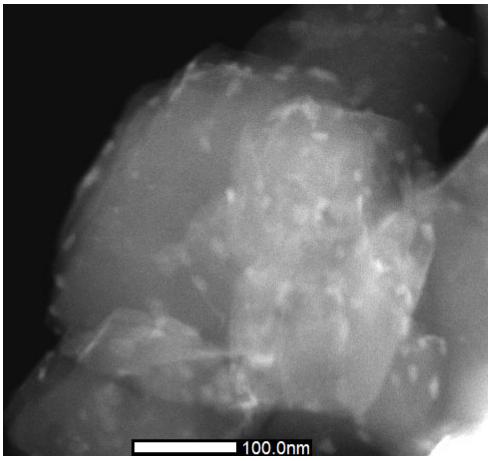

1TYN

Figure S4. STEM images of the samples with Fe, Co, Ni supported on Ti-containing zeolite Y.

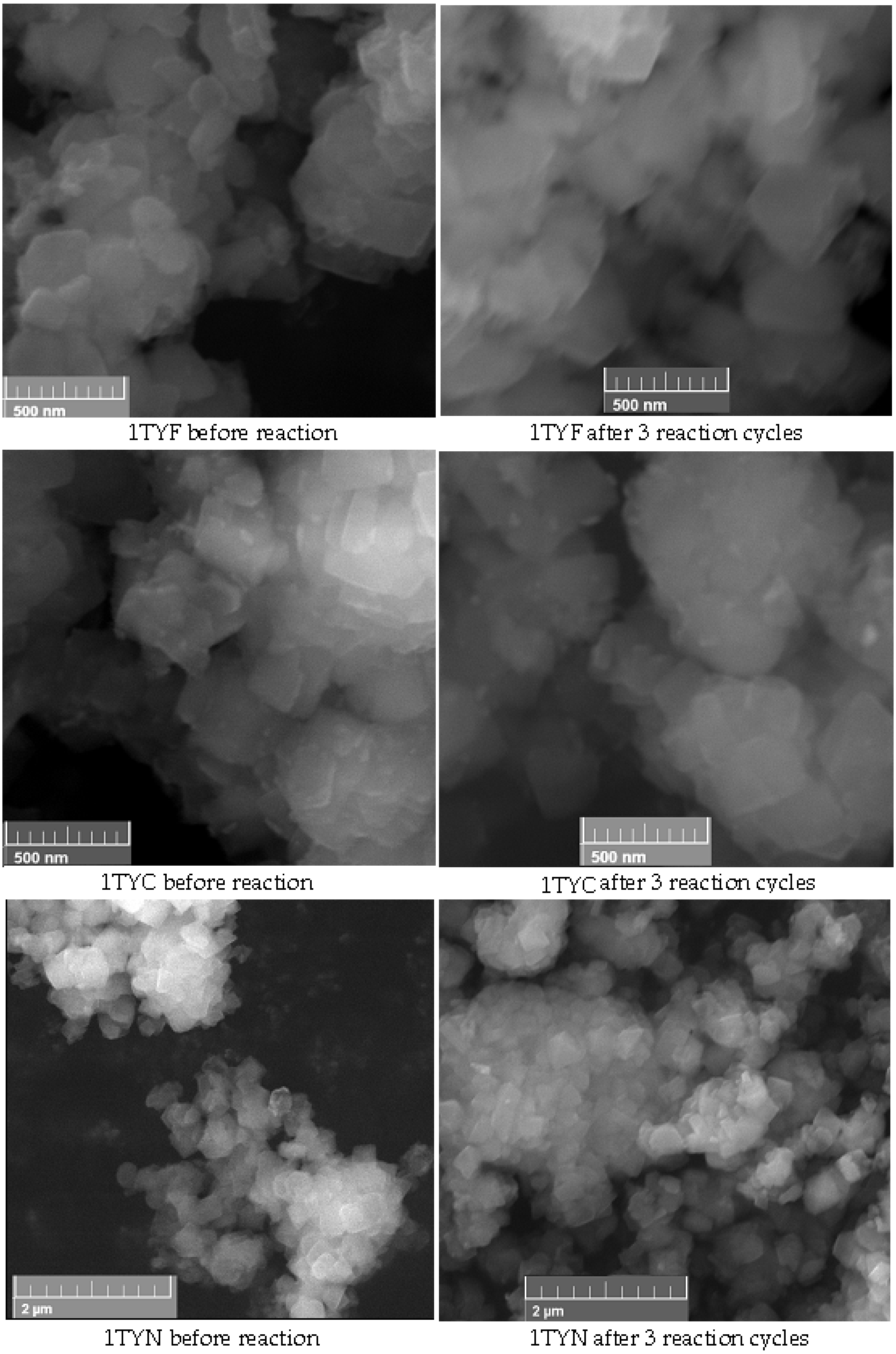

Figure S5. SEM images of the samples before and after photocatalytic reactions.

Table S1. EDX results.

Element	keV	1TYF		1TYC		1TYN	
		Weight, %	Atomic, %	Weight, %	Atomic, %	Weight, %	Atomic, %
O	0.525	62.62	47.41	59.75	44.40	57.59	41.74
Al	1.486	7.13	9.11	6.37	7.98	3.31	4.05
Si	1.739	27.72	36.84	31.44	41.01	35.75	45.47
Ti	4.508	0.16	0.37	0.15	0.33	0.32	0.70
Fe	6.398	2.37	6.27	-	-	-	-
Co	6.924	-	-	2.29	6.28	-	-
Ni	7.471	-	-	-	-	3.03	8.04
